# Association between adherence to the Mediterranean diet and the prevalence of cardiovascular risk factors

**DOI:** 10.1590/1518-8345.3904.3295

**Published:** 2020-06-08

**Authors:** Enrique Ramón-Arbués, Blanca Martínez-Abadía, José Manuel Granada-López, Emmanuel Echániz-Serrano, Isabel Huércanos-Esparza, Isabel Antón-Solanas

**Affiliations:** 1 Universidad San Jorge, Facultad de Ciencias de la Salud, Zaragoza, Esp, Espanha.; 2 MAS Prevención, Servicio de Prevención, Zaragoza, Esp, Espanha.; 3 Universidad de Zaragoza, Facultad de Ciencias de la Salud, Zaragoza, Esp, Espanha.; 4 Grupo de Investigación Tranfercult (Exp. H27-20D), Zaragoza, Esp, Spain.

**Keywords:** Diet, Mediterranean, Cardiovascular Diseases, Risk Factors, Cross-Sectional Studies, Workers, Occupational Health Nursing, Dieta Mediterrânea, Doenças Cardiovasculares, Fatores de Risco, Estudos Transversais, Trabalhadores, Enfermagem do Trabalho, Dieta Mediterránea, Enfermedades Cardiovasculares, Factores de Riesgo, Estudios Transversales, Trabajadores, Enfermería del Trabajo

## Abstract

**Objective:**

to determine the prevalence of cardiovascular risk factors in a cohort of workers and to quantify its association with compliance with the Mediterranean diet follow-up.

**Method:**

a cross-sectional descriptive study was carried out on a cohort of 23,729 workers. Clinical data from annual medical examinations and the Mediterranean Diet Adherence Screener were used to assess adherence to the Mediterranean diet.

**Results:**

51.3% of the participants showed good adherence to the Mediterranean diet. The multivariate analysis showed an inverse and significant association between the follow-up of the Mediterranean diet and the prevalence of abdominal obesity (Odds Ratio = 0.64, 95% CI 0.56; 0.73), dyslipidemia (Odds Ratio = 0.55, 95% CI 0.42; 0.73), and metabolic syndrome (Odds Ratio = 0.76, 95% CI 0.67; 0.86).

**Conclusions:**

our results suggest that the Mediterranean diet is potentially effective in promoting cardiovascular health. Implementing the interventions promoting the Mediterranean diet in the working population seems justified.

## Abstract (pt)

**Objetivo:**

determinar a prevalência de fatores de risco cardiovascular em uma coorte de trabalhadores e quantificar sua associação com o monitoramento da dieta mediterrânea.

**Método:**

estudo descritivo transversal, realizado em uma coorte de 23.729 trabalhadores. Dados clínicos dos exames médicos anuais e a *Mediterranean* Diet *Adherence Screener* foram usados para avaliar a adesão à dieta mediterrânea.

**Resultados:**

51,3% dos participantes apresentaram boa adesão à dieta mediterrânea. A análise multivariada mostrou associação inversa e significativa entre o acompanhamento da dieta mediterrânea e a prevalência de obesidade abdominal (*Odds Ratio* = 0,64, IC 95% 0,56, 0,73), dislipidemia (*Odds Ratio* = 0,55, IC 95% 0,42, 0,73) e de síndrome metabólica (*Odds Ratio* = 0,76, IC 95% 0,67, 0,86).

**Conclusões:**

nossos resultados sugerem que a dieta mediterrânea é potencialmente eficaz na promoção da saúde cardiovascular. A implementação de intervenções que promovem a dieta mediterrânea na população trabalhadora parece justificada.

## Abstract (es)

**Objetivo:**

determinar la prevalencia de factores de riesgo cardiovascular en una cohorte de trabajadores y cuantificar su asociación con el seguimiento de la dieta mediterránea.

**Método:**

se llevó a cabo un estudio descriptivo transversal sobre una cohorte de 23.729 trabajadores. Se utilizaron los datos clínicos procedentes de los exámenes médicos anuales y el *Mediterranean Diet Adherence Screener* para evaluar la adherencia a la dieta mediterránea.

**Resultados:**

el 51,3% de los participantes presentó una buena adherencia a la dieta mediterránea. El análisis multivariante evidenció una asociación inversa y significativa entre el seguimiento de la dieta mediterránea y la prevalencia de obesidad abdominal (*Odds Ratio* = 0,64, IC 95% 0,56; 0,73), dislipidemia (*Odds Ratio* = 0,55, IC 95% 0,42; 0,73) y de síndrome metabólico (*Odds Ratio* = 0,76, IC 95% 0,67; 0,86).

**Conclusión:**

nuestros resultados sugieren que la dieta mediterránea es potencialmente eficaz en la promoción de la salud cardiovascular. Parece justificada la implementación de intervenciones que promuevan la dieta mediterránea en la población trabajadora.

## Introduction

Cardiovascular (CV) diseases represent the leading cause of death worldwide. Every year more than 17 million people die from this cause, which represents more than 30% of the total deaths registered^(^ 1 ^)^. In Spain, these diseases rank third in terms of potential years of life lost, only behind tumors and external causes^(^ 2 ^)^. From the epidemiological point of view, it is estimated that the action of factors traditionally linked to them may lead to a significant reduction in morbidity and mortality^(^ 3 ^-^ 4 ^)^. In particular, it is considered that diet may be the behavioral factor with the greatest impact in minimizing CV risk^(^ 5 ^)^. Among the dietary patterns that reduce CV morbidity and mortality, the Mediterranean diet (MedDi) has been pointed out. This diet is based on a generous intake of fruits, vegetables, complex carbohydrates or mono-unsaturated fats and, on the contrary, a low intake of animal fats and sugars. Its preventive mechanism would be supported by its positive influence on blood pressure, body weight, glycemic control, vascular inflammation or arteriosclerosis, among others^(^ 6 ^)^.

Several studies have analyzed the prevalence of CV risk factors in the Spanish population^(^ 7 ^-^ 9 ^)^. However, these investigations have been carried out on the general population, with little literature on the prevalence of these factors in more specific populations such as workers. Similarly, there are still gaps in the literature regarding the association between MedDi follow-up and the prevalence of CV risk factors motivated by the great heterogeneity of the studies and their poor methodological quality^(^ 10 ^)^. Based on these deficiencies, the objectives of this investigation were to determine the prevalence of CV risk factors in a cohort of workers from Aragon (Spain) and to quantify its association with the MedDi follow-up.

## Method

A cross-sectional descriptive study was carried out. Prior to the start of the investigation, the authorization of the Ethics Committee for Clinical Research in Aragon was requested. At all times, the confidentiality of the data established by the current Organic Law on Data Protection was respected. In addition, all the participants signed an informed consent after being informed of the objectives and methodology of the study.

The study population was that of workers in the Aragon region with health surveillance arranged with *MAS-Prevención*, a company for preventing occupational risks with major presence throughout Spain. The participants were recruited consecutively in the company medical exams sessions implemented by *MAS-Prevención* during the first half of 2018.

The workers who agreed to participate in the survey were 25,613; 763 were excluded from the analysis after showing a fast of less than 10 hours, 459 due to problems derived from the management of blood samples and 662 due to omissions or possible errors in data transcription. Thus, the final sample size was 23,729.

The participants were asked for information regarding their age, relationship with tobacco and alcohol, physical activity (PA) and adherence to the MedDi. Subsequently, according to the standardized protocol of medical examination of *MAS-Prevención*, trained health personnel examined the participants (blood pressure, weight, height and abdominal circumference) and performed a blood draw.

Alcohol consumption (in grams - gr) was calculated based on the following formula: $\mathrm{Grams}=\left[\mathrm{graduationXvolume}\;\left(\mathrm{cl}\right)\;X0.8\right]/100$. From this calculation, a daily intake > 30 g in men and > 20 g in women^(^ 11 ^)^ was considered as risky alcohol consumption.

All the participants who did not comply with any of the activity recommendations described by the World Health Organization (WHO) for people aged 18 to 64 years old were considered inactive/sedentary^(^ 12 ^)^.

The Mediterranean Diet Adherence Screener (MEDAS-14) was used to assess the degree of adherence to the MedDi. This is a specific questionnaire of fourteen items validated in the general population^(^ 13 ^)^. To obtain your score, the value +1 is assigned to each item with a positive connotation with respect to the MedDi. The degree of adherence is determined from the sum of the obtained values, establishing two levels: Acceptable MedDi adherence (score ≥9), and low adherence (<9).

Blood pressure was assessed with the patient in the supine position and the arm at the level of the heart; three measurements were made with 1-minute intervals between them. The reported blood pressure was the mean of the three determinations. Arterial hypertension (AHT) was considered in view of systolic blood pressure values ≥140 mmHg and/or diastolic blood pressure ≥90 mmHg.

The height of the participants was determined, without footwear, using an approved portable stadiometer (model SECA 213^©^). The participants were weighed in light underwear using an approved electronic scale (Detecto PD200^©^). The measurement of the abdominal perimeter (in centimeters - cm) was carried out, with the participant in a standing position, using a tape measure placed parallel to the ground and covering the contour between the upper part of the iliac crests and the lower rib. A risky abdominal perimeter (central/abdominal obesity) was considered when the measurements were ≥88 cm in women and ≥102 cm in men^(^ 14 ^)^.

For the blood test, the participant must have respected a night fast of, at least, 10 hours. The samples were sent to the entity’s laboratory (accredited by the National Accreditation Entity). All the participants with baseline blood glucose ≥126 mg/dl (or under anti-diabetic treatment) were classified as diabetic, and those who reported being under pharmacological treatment for lipid alterations or met any of the diagnostic criteria of the Spanish Society of Arteriosclerosis were considered as dyslipidemic^(^ 15 ^)^. Finally and based on international consensus criteria^(^ 16 ^)^, the existence of metabolic syndrome was evaluated.

The descriptive analysis of the characteristics of the sample was carried out using the mean and standard deviation for the quantitative variables and the number and percentage for the qualitative ones. The comparison by sex of these characteristics was performed using the Student’s t-test on the quantitative variables and the Chi-square for the qualitative ones. The analysis of the relationship between compliance with the MedDi and the presence of CV risk factors was performed using different binary logistic regression models (forward Wald method). The results of these models are presented through the Odds Ratio (OR) and 95% confidence intervals with adjustment for age, sex and other factors potentially associated with the development of diabetes, dyslipidemia, AHT, obesity and/or metabolic syndrome indicated by the previous bibliography^(^ 17 ^-^ 20 ^)^. All the calculations were performed with the SPSS Version 21.0 software, accepting the significance level of α≤0.05.

## Results

The mean age of the participants was 42.5 years old. The body mass index (BMI), blood pressure, blood glucose, total cholesterol, low density lipoprotein(LDL) and triglycerides were higher in the men’s group (p<0.001). In contrast, high density lipoprotein (HDL), cholesterol levels were higher in women. The prevalence of smoking, physical inactivity, central obesity, and overweight/obesity were 31.5%, 56.5%, 25.3%, and 55.3%, respectively. The prevalence of hypertension was 20.1%, that of dyslipidemia was 31.3%, and that of metabolic syndrome was 7.5%. In all these cases, the prevalences in the men’s group were significantly higher than those observed in the women’s group (p<0.001). In contrast, abdominal obesity was more frequent among women (30.2% vs. 23.1%). There were no significant differences between the sex in the prevalence of diabetes (Table 1).

**Table 1:** Sociodemographic, anthropometric and analytical characteristics of the participants (n=23,729). Mean ± standard deviation or number (percentage). Aragon Region, AR, Spain, 2018

Variables	Total (n=23,729)	Females (n=7,693)	Men (n=16,036)	p
Age (years old)	42.5±10.3	41.85±10.2	42.91±10.4	0.114
16-25 years old	1059 (4.5%)	362 (4.7%)	697 (4.3%)	<0.001
26-35 years old	5354 (22.6%)	1867 (24.3%)	3487 (21.7%)	
36-45 years old	7954 (33.5%)	2646 (34.4%)	5308 (33.1%)	
46-55 years old	6447 (27.2%)	2002 (26.0%)	4445 (27.7%)	
56-65 years old	2915 (12.3%)	816 (10.6%)	2099 (13.0%)	
Weight (kilograms - kg)	76.6±15.8	65.03±12.9	82.21±13.9	<0.001
BMI (kg/m^2^)*	26.1±4.5	24.56±4.7	26.91±4.1	<0.001
Low weight (<18.5 kg/m^2^)*	388 (1.6%)	300 (3.9%)	88 (0.5%)	<0.001
Normal weight (18.5-24.9 kg/m^2^)*	9996 (42.1%)	4528 (58.9%)	5468 (34.1%)	
Overweight (25-29.9 kg/m^2^)*	9153 (38.6%)	1911 (24.8%)	7242 (45.2%)	
Type I obesity (30-34-9 kg/m^2^)*	3221 (13.6%)	671 (8.7%)	2550 (15.9%)	
Type II obesity (35-39-9 kg/m^2^) *	758 (3.2%)	211 (2.7%)	547 (3.4%)	
Abdominal perimeter (centimeters)	90.1±15.0	82.57±12.1	93.7±4.9	<0.05
Abdominal obesity	6020 (25.3%)	2320 (30.2%)	3700 (23.1%)	<0.001
Diastolic blood pressure (mmHg)^†^	78.89±12.3	77.21±11.8	79.26±11.9	<0.001
Systolic blood pressure (mmHg)^†^	133.16±17.6	127.23±16.7	135.41±17.8	<0.001
Arterial hypertension	4773 (20.1%)	1163 (15.1%)	3610 (22.5%)	<0.001
Total cholesterol (mg/dl)^‡^	191.6±36.0	188.77±34.0	193.0±36.8	<0.001
HDL cholesterol (mg/dl)^‡^	60.9±15.5	70.68±15.5	56.33±13.2	<0.001
LDL cholesterol (mg/dl)^‡^	108.2±31.1	101.00±29.2	111.74±31.4	<0.001
Triglycerides (mg/dl)^‡^	113.9±82.2	85.59±42.5	127.56±92.5	<0.001
Total cholesterol/HDL cholesterol	3.31±0.9	2.77±0.6	3.57±0.97	<0.001
Dyslipidemia	7430 (31.3%)	1707 (22.2%)	5723 (35.7%)	<0.001
Glycaemia (mg/dl)^‡^	89.1±17.1	85.17±12.7	91.08±18.5	<0.001
Diabetes Mellitus	1824 (7.6%)	598 (7.0%)	1226 (7.6%)	0.729
Metabolic syndrome	1795 (7.5%)	533 (6.9%)	1262 (7.9%)	<0.05
Risky alcohol consumption	233 (1.0%)	49 (0.6%)	184 (1.1%)	<0.001
Smoking	7488 (31.5%)	2083 (27.1%)	5405 (33.7%)	<0.001
Physical inactivity	13425 (56.5%)	3978 (51.7%)	9447 (58.9%)	<0.001

* kg/m^2^= kilograms per square meter;

† mmHg = millimeters of mercury;

‡ mg/dl = milligrams *per* deciliter

The participants’ age was correlated with higher prevalences of abdominal obesity, overweight/obesity, diabetes, AHT and metabolic syndrome; 51.3% of the participants showed an acceptable adherence to the MedDi. The components of the scale with the highest degree of compliance were the preferred use of olive oil, the preferred consumption of white over red meat and the consumption of 2 or more servings of vegetables *per* day. In contrast, the lowest levels of compliance were those related to the consumption of red wine, legumes, nuts, commercial pastries and fish. In the sex analysis, women showed higher consumptions of olive oil, fish and commercial pastries. Men consumed more nuts, wine, sugary/carbonated drinks and animal fats (margarine, butter, cream, etc.) (Table 2).

**Table 2:** Results by sex of the MEDAS-14 questionnaire*, expressed in number and percentage (n=23,729). Aragon Region, AR, Spain, 2018

Criterion	Total (n=23,729)	Females (n=7,693)	Men (n=16,036)	p
Using olive oil as the main source for cooking fats	20882 (88.0%)	7023 (91.3%)	13859 (86.4%)	<0.001
Daily consumption of 2 or more tablespoons of olive oil	15423 (64.9%)	5062 (65.8%)	10361 (64.6%)	0.072
Daily consumption of 2 or more servings of vegetables	13787 (58.1%)	4477 (58.2%)	9310 (58.0%)	0.839
Daily consumption of 3 or more pieces of fruit (includes natural juice)	10986 (46.3%)	3558 (46.2%)	7428 (46.3%)	0.918
Daily consumption less than 1 serving of red meats, sausages or cold meats	13194 (55.6%)	4292 (55.8%)	8902 (55.5%)	0.686
Daily consumption less than 1 serving of butter, margarine or cream	12625 (53.2%)	4392 (57.1%)	8233 (51.3%)	<0.001
Daily consumption less than 1 carbonated and/or sugary drink	12123 (51.1%)	4084 (53.0%)	8039 (50.1%)	<0.001
Weekly consumption of 3 or more glasses of red wine	4366 (18.3%)	623 (8.1%)	3743 (23.3%)	<0.001
Weekly consumption of 3 or more servings of legumes	5601 (23.6%)	1869 (24.3%)	3732 (23.2%)	0.083
Weekly consumption of 3 or more servings of fish	8946 (37.7%)	3085 (40.1%)	5861 (36.5%)	<0.001
Weekly consumption less than 3 servings of commercial pastries	7461 (31.4%)	2492 (32.4%)	4969 (31.0%)	<0.05
Weekly consumption of 1 or more servings of nuts	5434 (22.9%)	1654 (21.5%)	3780 (23.5%)	<0.001
Preferred consumption of white over red meat	13739 (57.9%)	4501 (58.5%)	9238 (57.6%)	0.189
Weekly consumption of 2 or more dishes garnished with vegetable sauce and olive oil	12718 (53.6%)	4161 (54.1%)	8557 (53.3%)	0.293
Final score ≥9 points^†^	12178 (51.3%)	3992 (51.9%)	8186 (51.0%)	0.224
Final Score. Mean±Standard Deviation^†^	9.10±3.98	9.18±4.09	9.06±3.92	<0.05

* MEDAS-14 = Mediterranean Diet Adherence Screener;

† Final score. Compliance with the criterion equals 1 point and non-compliance equals 0

The adjusted multivariate analysis showed an inverse and significant relationship between the acceptable follow-up of the MedDi and the prevalence of central obesity [Odds Ratio-OR (95% CI) = 0.64 (0.56, 0.73)], dyslipidemia [OR (95% CI) = 0.55 (0.42, 0.73)] and metabolic syndrome [OR (95% CI) = 0.76 (0.67, 0.86)]. A different result was obtained when examining the relationship between the MedDi and the prevalence of diabetes, AHT, and overweight/obesity. The behavior of the groups under study (total sample, women and men) was analogous (Table 3).

**Table 3:** Odds Ratio [95% Confidence Interval] adjusted for CV risk factors in relation to acceptable adherence to the Mediterranean diet (MEDAS-14.*≥9) Aragon Region, AR, Spain, 2018

	Total (n=23,729)	Women (n=7,693)	Men (n=16,036)
Overweight/Obesity^†^	0.96 (0.88; 1.05)	0.93 (0.85; 1.02)	0.98 (0.91; 1.06)
Central obesity^†^	0.64 (0.56; 0.73)^§^	0.61 (0.51; 0.72)^§^	0.65 (0.55; 0.76)^§^
Diabetes^‡^	0.93 (0.84; 1.03)	0.91 (0.78; 1.06)	0.88 (0.71; 1.09)
Hypertension^‡^	0.89 (0.78; 1.02)	0.91 (0.8; 1.04)	0.89 (0.77; 1.01)
Dyslipidemia^‡^	0.55 (0.42; 0.73)^§^	0.78 (0.69; 0.87)^§^	0.82 (0.72; 0.93)^§^
Metabolic syndrome^‡^	0.76 (0.67; 0.86)^§^	0.73 (0.59; 0.91)^§^	0.78 (0.67; 0.90)^§^

* MEDAS-14 = Mediterranean Diet Adherence Screener;

† Model adjusted for age, gender, smoking, level of physical activity - PA - and alcohol consumption;

‡ Model adjusted for age, gender, smoking, PA level, alcohol consumption, overweight/obesity and central obesity;

§ p<0.05

## Discussion

The results of this research show a working population with a high prevalence of CV risk factors. However, the figures obtained are below the bulk of those reported in other Spanish population-based studies^(^ 2 ^,^ 7 ^-^ 9 ^,^ 17 ^)^. This difference may be related to the location of the different studies. The sample of this study lies in the northeast of Spain, far from the southern area of the country where higher prevalences of CV risk factors have traditionally been reported and, in general, a worse socioeconomic situation^(^ 21 ^)^. In any case, this comparison and its interpretation should be taken with caution, due to the differences in the baseline characteristics of the participants of the various studies (especially age groups) and in the diagnostic criteria of certain CV risk factors (dyslipidemia and metabolic syndrome). The literature on the prevalence of CV risk factors in Spanish workers is very scarce. A study was found, started in 2009 but still under follow-up, where 80.3%, 47.1%, 40.0% and 7.4% prevalences of overweight/obesity, hypertension, smoking and diabetes were reported, respectively^(^ 22 ^)^.

As for our participants, 51.3% reported acceptable adherence to the MedDi. At this point, the results of previous studies are very heterogeneous, with adherence ranges between 14% and 58.3%^(^ 23 ^-^ 28 ^)^. The relatively privileged situation of this sample compared to that of previous studies could be due to the intense Worker Health Promotion strategy implemented by *MAS-Prevención* in the last 5 years. This strategy, fundamentally implemented by the institution’s work nurses, is based on a powerful educational commitment to healthy lifestyles.

In any case, the comparison of the results of this study with the previous bibliography is complex given the differences in the populations analyzed and the use of different questionnaires to assess adherence to the MedDi. In this sense, a recent review study^(^ 29 ^)^ identifies up to 28 screening tools for MedDi adherence and defends the need to develop a questionnaire with greater conceptual and methodological rigor.

The participants in this study evidenced a significant and inverse association between MedDi adherence and the prevalence of abdominal obesity, dyslipidemia, and metabolic syndrome. In the previous literature on the theme, a great variability was observed in the results in relation to this association. In order to clarify these divergences, several secondary studies have been carried out in recent years. In a Cochrane meta-analysis^(^ 30 ^)^ of the MedDi-based intervention studies, significant improvements in total cholesterol and LDL values were demonstrated. However, the available evidence was considered insufficient to draw conclusions about the association between the MedDi and blood pressure. In 2016, a meta-analysis of studies based on MedDi-based interventions also revealed inverse and significant associations between this dietary pattern and the levels of triglycerides, glycaemia, blood pressure, and abdominal circumference^(^ 31 ^)^. Finally, in a meta-analysis of observational studies from 2017, a lower probability of suffering central obesity, hypertension and metabolic syndrome was deduced in people with good adherence to the MedDi^(^ 32 ^)^.

This study presents several limitations that may affect the generalization of our results. The population consisted of workers, therefore the findings may not be extrapolated to non-active people. Likewise, it is made up mostly of men (67.5%) who may be representative of the working population of the region but not of the general population. Regarding the association between MedDi monitoring and the prevalence of CV risk factors, another two limitations should be noted. The first is the cross-sectional design of the study, which allows establishing associations but not cause-effect relationships. The second responds to the dichotomization of the nutrition variable based on the monitoring of the MedDi. In this way, other possible dietary patterns that, in one way or another, could qualify the association are excluded from the analysis. Therefore, it is suggested that new research studies be launched to clarify the relationship between MedDi monitoring and the prevalence of CV risk factors, which have longitudinal designs and incorporate, in their analysis, a wider range of eating patterns.

To the best of our knowledge, this is the first investigation evaluating the association between MedDi adherence and the prevalence of each of the classic non-behavioral CV risk factors (diabetes, dyslipidemias, hypertension, obesity, and metabolic syndrome) in the same population (particularly workers). Apart from the novelty, the main strengths of this study are the large sample size, the standardized data collection procedures (anthropometric, clinical, and laboratory), and the biological plausibility of the different observed associations. All this allows us to establish a reliable map of the CV health and habits of the working population of Aragon (Spain), which can serve as a starting point for the development of strategies (preventive and diagnostic) and health programs.

## Conclusion

The results of this study show an active population subjected to a high exposure to CV risk factors. This circumstance suggests that the incidence of CV morbidity and mortality will progressively increase in the population. In addition, the low adherence to the MedDi of the working population of Aragon and the inverse association observed between their follow-up and the prevalence of CV risk factors are noteworthy. Under this prism it seems necessary to implement new social and health policies aimed at promoting a balanced eating pattern such as the MedDi. In this sense, occupational nurses, health surveillance professionals with close proximity and ancestry over the workers, must remain natural leaders.
